# Continuous Readout versus Titer-Based Assays of Influenza Vaccine Trials: Sensitivity, Specificity, and False Discovery Rates

**DOI:** 10.1155/2019/9287120

**Published:** 2019-05-08

**Authors:** Dongmei Li, Jiong Wang, Jessica Garigen, John J. Treanor, Martin S. Zand

**Affiliations:** ^1^Informatics Core, Clinical and Translational Science Institute, University of Rochester Medical Center, Rochester, NY, USA; ^2^Department of Medicine, Division of Nephrology, University of Rochester Medical Center, Rochester, NY, USA; ^3^Department of Medicine, Division of Infectious Diseases, University of Rochester Medical Center, Rochester, NY, USA; ^4^Rochester Center for Health Informatics, University of Rochester Medical Center, Rochester, NY, USA

## Abstract

The current gold standard for measuring antibody-based immunity to influenza viruses relies on the hemagglutinin inhibition assay (HAI), an 80-year-old technology, and the microneutralization assay (MN). Both assays use serial dilution to provide a discrete, ranked readout of 8–14 categorical titer values for each sample. In contrast to other methods of measuring vaccine antibody levels that produce a continuous readout (i.e., mPLEX-Flu and ELISA), titering methods introduce imprecision and increase false discovery rates (FDR). In this paper, we assess the degree of such statistical errors, first with simulation studies comparing continuous data with titer data in influenza vaccine study group comparison analyses and then by analyzing actual sample data from an influenza vaccine trial. Our results show the superiority of using continuous, rather than discrete, readout assays. Compared to continuous readout assays, titering assays have a lower statistical precision and a higher FDR. The results suggested that traditional titering assays could lead to increased Type-II errors in the comparison of different therapeutic arms of an influenza vaccine trial. These statistical issues are related to the mathematical nature of titer-based assays, which we examine in detail in the simulation studies. Continuous readout assays are free of this issue, and thus it is possible that comparisons of study groups could provide different results with these two methods as we have shown in our case study.

## 1. Introduction

Both seasonal and emerging influenza virus infections constitute one of the largest global public health threats [1]. The influenza virus has two major viral surface glycoproteins, hemagglutinin (HA) and neuraminidase (NA), both of which can induce a strong humoral immune response [2]. On the basis of antibody serotypes and genotypes, 18 HA subtypes and 11 NA subtypes are currently recognized within the known influenza A virus strains [3]. Antibodies against the HA of influenza are essential for protection against influenza virus infection [4]. Previous studies reported that preexisting IgG antibodies from previous infection or vaccination in childhood could affect the generation and maintenance of homologous and cross-reactive antibodies against influenza viruses. This phenomenon has been variously termed “original antigenic sin” (OAS), antigenic seniority, or HA imprinting [5–8]. Even the most recent studies of OAS have indicated that the antibody responses against individual influenza strains are hierarchical and are determined by the first and subsequent influenza infections in childhood [7, 9]. However, the question of how preexisting antibodies affect the B-cell response against either subsequent influenza infections or vaccines, especially cross protection against current influenza viruses with antigenic drift or shift in every flu season, still remains obscure. Moreover, understanding the immunological mechanism of either OAS or HA imprinting is critical for developing new vaccines.

The motivation for this study lies in potential flaws with two assays, considered standards, widely used to measure anti-HA antibody activity and protection in clinical trials; these are the hemagglutinin inhibition (HAI) assay [10–12] and the microneutralization (MN) assay [13, 14]. Both assays are semiquantitative, providing only a discrete ranked readout of one of 8–14 titer values based on two-fold dilutions of serum samples (i.e., 1 : 10,1 : 20,1 : 40,   …, 1 : 2560). In these assays, the result is the highest dilution of the test sample resulting in positive tests. This titer value is subject to round-off error, in that all potentially positive dilutions above the titer value are effectively rounded down. For example, when testing dilutions 1 : 20 and 1 : 40, there is no possibility of finding an intermediate value (e.g., 1 : 30). This can result in both inflated Type-I (probability of having false positives, 1 − specificity) and Type-II (probability of having false negatives, 1 − sensitivity) errors when estimating influenza antibody levels. Because of these potential errors, the likelihood of missing some significant differences between influenza vaccine treatment cohorts in clinical studies would be high. One solution to this problem lies in recently developed assays with a continuous readout (e.g., IgG concentrations). We have developed the mPLEX-Flu assay, a Luminex-based multiplex assay that simultaneously measures IgG antibody reactivity against up to 50 influenza strains/substrain HA proteins with only 1–5 *µ*L of serum, generating a continuous readout across a 4 log range [15–19].

To our knowledge, there are no prior reports in the literature directly comparing the results of titer-based (semiquantitative) and continuous readout assays on an experimental and theoretical basis to characterize the risk of such errors, especially their effects on subsequent treatment group comparisons in vaccine clinical trials. In this work, we report such a comparison, first using simulation studies and then with a directly comparing results from a clinical trial of H5 influenza vaccination [20]. This approach is advantageous as simulation studies can provide detailed information regarding sensitivity, specificity, and false discovery rates (FDR) obtained from statistical treatment group comparisons when comparing different treatment groups in vaccine clinical trials. Application to actual clinical trial data provides context for assessing whether any new between-group effects found to be statistically significant may be biologically or clinically significant.

In this manuscript, we first report results from simulation studies comparing vaccine treatment groups demonstrating the superiority of continuous assay data, as opposed to semiquantitative titer-based data, with respect to higher sensitivity, higher specificity, and lower FDR. A similar finding is reported when comparing semiquantitative (HAI and MN) and continuous (mPLEX-Flu) assay results from a clinical study of H5 influenza vaccination (DMID 08-0059) when using linear mixed models on log-transformed measurements. The mPLEX-Flu assay data revealed several statistically significant differences between study cohorts; these were not significant from analyses using the HAI assay and MN assay data. Our findings strongly suggest that the continuous readout multiplex method would likely detect more significant differences between vaccine groups than would titering assays. Such methods will be an improvement over current standards for characterizing pre- and postvaccine IgG-mediated immunity against influenza viruses and also the influence of previous influenza vaccination on the antibody response.

## 2. Methods

### 2.1. Human Subjects Ethics Statement

This study was approved by the Research Subjects Review Board at the University of Rochester Medical Center (RSRB approval number RSRB00012232). Clinical samples were analyzed under secondary use consent obtained previously as part of a prior clinical trial [20]. Written informed consent was obtained from all participants and maintained as per RSRB regulations. Research data were coded such that subjects could not be identified, either directly or through linked identifiers, in compliance with the Department of Health and Human Services' Regulations for the Protection of Human Subjects (45 CFR 46.101(b) (4)).

### 2.2. Samples and Data

Serum samples for the multiplex assay were obtained from a prior clinical trial, DMID 08-0059 (Table 1) [20]. The subjects who missed before vaccination (day 0) baseline were excluded, and all data (mPLEX-Flu, HAI, and MN) were adjusted by dose difference with linear mixed effects models. All subjects in the three cohorts were inoculated with inactivated A/Indonesia/5/05 (A/Ind05) vaccine. Primed subjects (*n*=46) previously received the inactivated subvirion vaccine against influenza A/Vietnam/1203/04 (A/Vie04) in 2005-2006. The multiple boost group (*n*=16) had received both the recombinant influenza A/Hong Kong/156/97 vaccine (A/HK97) in 1997-1998 and the influenza A/Vie04 vaccine in 2005-2006. Unprimed subjects, i.e., H5-naive subjects (*n*=31), received 2 identical A/Ind05 vaccinations separated by 28 days. Blood samples were collected before vaccination (day 0) and on days 7, 14, 28, 56, and 180 after vaccination. Blood samples were also collected from the unprimed subjects on days 7, 14, and 28 after the second immunization.

### 2.3. mPLEX-Flu Analysis

We measured the concentrations of anti-HA IgG antibodies against recombinant HA from 45 strains of influenza viruses in serum samples previously gathered in the DMID 08-0059 study [20] by using the mPLEX-Flu assay [15]. The calculation of individual IgG concentrations for each influenza strain anti-HA IgG was performed using standard curves generated from five-parameter logistic regression models [21]. All recombinant HA (rHA) proteins were full length trimers. In this study, we focused on the homologous antibodies against three H5 vaccine strains, A/Hong Kong/157/1997 (HK97), A/Vietnam/1203/2004 (Vie05), and A/Indonesia/5/2005 (Ind05). All data generated by the mPLEX-Flu assay are contained in the Supplemental Material S1 data file. Linear mixed effects models with group, day, and group-day interaction were used to fit the data for each H5 vaccine strain. Covariates adjusted in the linear mixed effects models included the following: age at enrollment, gender, ethnicity (Caucasian vs. non-Caucasian), dose (two dose levels: 15 and 90 *µ*g), and batch (five batches).

### 2.4. Reanalyses of HAI and MN Data

All HAI and MN data were generated during the DMID 08-0059 study, as previously described [20]. Serum antibody responses to the homologous A/Indonesia/05/2005 virus were measured at the Southern Research Institute, as previously described [22]. The neutralizing antibody response was measured by microtiter neutralization of influenza virus added to cultures of Madin-Darby canine kidney cells to measure the viral protein level after 18 hours' infection [23]. HAI assays were performed with horse erythrocytes as indicator cells using the WHO standard assay protocol [22]. All serum samples were tested at a starting dilution of 1 : 10, with negative results assigned a titer of 5 for calculation purposes. The replicate geometric mean was calculated to determine the antibody titer for each sample. We reanalyzed those data using linear mixed effects models, with repeated measurements on the same strain taken into account [24]. The same predictors and covariates were used in the linear mixed effects models for the HAI and MN data analysis as were used for the mPLEX-Flu data analysis. Data are available in the Supplementary Materials S2 data file.

### 2.5. Computational Environment

All HAI and MN data from the DMID 08-0059 study were stored on a secure LabKey Server [20]. Serum anti-HA IgG concentrations were estimated using the mPLEX-Flu assay, as described above. A standard curve was fitted to the mean fluorescence intensity (MFI) results on log_2_ scale for each HA using the five-parameter logistic regression model, as follows:

(1) $$\begin{array}{c} f\left(x_{i},\theta \right)=\theta_{2}+\frac{\theta_{3}-\theta_{2}}{{\left(1+{\left(x_{i}/\theta_{4}\right)}^{\theta_{1}}\right)}^{\theta_{5}}}, \end{array}$$

where *x*_*i*_ is the dilution level in log_2_ or log_10_ scale, with *θ*_2_ being the minimum response, *θ*_3_ denoting the maximum response, *θ*_4_ denoting the concentration that results in 50% response, *θ*_1_ being the relative slope around the 50% response, and *θ*_5_ denoting the asymmetry in the dose-response relationship. After the individual HA standard curves were fitted using the five-parameter logistic regression model, they were used to estimate absolute anti-HA IgG concentrations from the mPLEX-Flu MFI values. We then log transformed the estimated concentration data, giving an approximate normal distribution. For this reason, the simulation studies were conducted based on the normal distribution with the range of data consistent with the real samples.

## 3. Results

### 3.1. The mPLEX-Flu Assay Is Highly Correlated with the HAI and MN Assays

**Figure 1:**
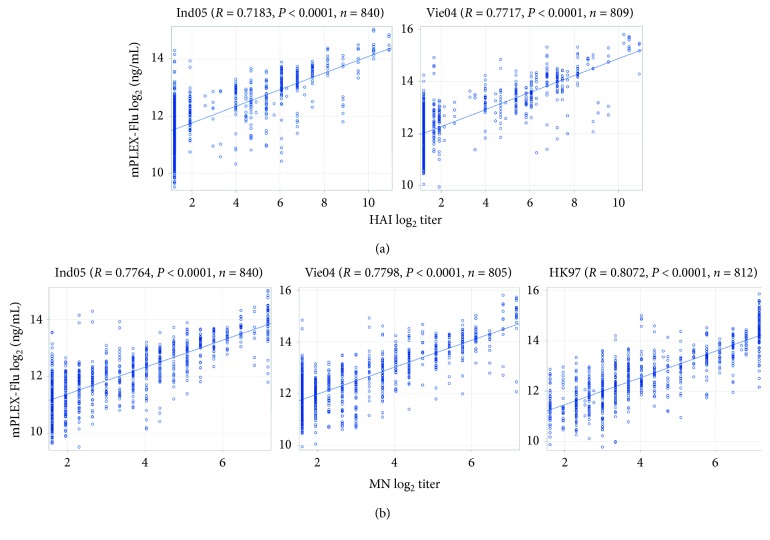
The correlation of mPLEX-Flu concentrations with HAI and MN titers. (a) Correlation of mPLEX-Flu concentrations with HAI titers for A/Indonesia/05/2005 (Ind05) and A/Vietnam/1203/2004 (Vie04). (b) Correlation of mPLEX-Flu concentrations with MN titers for A/Indonesia/05/2005 (Ind05), A/Vietnam/1203/2004 (Vie04), and A/HongKong/156/1997 (HK97).

Both HAI and MN assays are semiquantitative, but until now, they are still considered the gold standard assays for estimating anti-influenza virus antibody concentrations. We therefore began by examining the correlation of the mPLEX-Flu assay with HAI and MN assays. We calculated Pearson's correlation coefficients using pairwise comparisons of the mPLEX-Flu assay results (absolute IgG concentrations) against A/Indonesia/05/2005 (Ind05), A/Vietnam/1203/2004 (Vie04) from HAI and Ind05, Vie04, and A/HongKong/156/1997 (HK97) data from MN assay using the data from the DMID 08-0059 study [20]. The analyses show that the mPLEX-Flu assay results are highly correlated with the titers obtained from the HAI (Figure 1(a)) and MN assays (Figure 1(b)), with all *P* ≤ 0.0001. Notably, the mPLEX-Flu assay concentrations appear to have a greater correlation with MN titers (*r* ^2^=0.7764 − 0.8072) than HAI titers (*r* ^2^=0.7183 − 0.7717). It is important to note that one would not expect a perfect correlation when comparing a continuous versus a categorical assay due to the effect of binning a continuous assay result. Thus, these *r* ^2^ values are quite significant for this type of comparison.

### 3.2. Motivating Question for Simulation Studies

The motivating question for this study was as follows: is there a difference in the statistical conclusions regarding comparative vaccine efficacy across different vaccine groups when data from categorical, semiquantitative titering (e.g., HAI and MN) versus continuous readout (e.g., mPLEX-Flu) assays are used for analysis of influenza vaccine clinical trials? The semiquantitative HAI and MN titering assays are currently used in influenza vaccine clinical trials to compare treatment groups. If these have lower sensitivity and specificity and a higher level of Type-II errors (i.e., rejecting the hypothesis that there is a difference between treatment groups), compared to a continuous readout assay (e.g., mPLEX-Flu), this would suggest that clinical trials should use continuous readout assays. To answer this question, we first explored possible differences using a simulated dataset in which the titering assay results were derived from a continuous simulated dataset. Simulation studies were conducted to examine the sensitivity and specificity of testing group differences using concentration data from the mPLEX-Flu assay versus the titer data from the HAI assay and the MN assay. The FDRs using data from different assays were also examined.

### 3.3. Simulation Description

**Figure 2:**
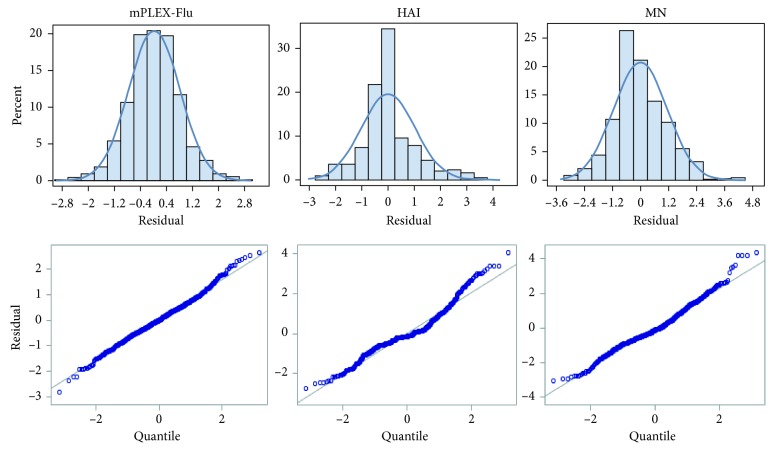
Residual histogram and Q-Q plot from the linear mixed effects model on the anti-influenza A/Indonesia/5/05 vaccine strain IgG results using the concentration data.

Based on the distribution of residuals from analyzing the log-transformed influenza vaccine data, we chose the multivariate normal distribution as the distribution for vaccine data in our simulation studies (Figure 2). Thus, we assumed that the logarithm of the measured IgG antibody reactivity levels *y*_*ijk*_ from the mPLEX-Flu assay for *i*th influenza virus strain, *j*th group, and *k*th sample (*i*=1,2,…, 100; *j*=1,2; *k*=1,2,…, *n*) follows a multivariate normal distribution, with a mean vector of *μ* (denoting the true logarithm of the IgG antibody reactivity levels) and a variance-covariance matrix of Σ, i.e.,

(2) $$\begin{array}{c} y_{ijk}∼\text{MVN}\left(\mu ,\Sigma \right). \end{array}$$

Among the 100 influenza strains in our simulation study, we assumed that there were different IgG antibody reactivity levels to the first *s* strains between the two groups. In the simulation studies, we set *s*=100 × *π*_1_ (*π*_1_=0.25, 0.30, 0.40, 0.50, 0.60, 0.75, 0.90) to cover different scenarios, with *π*_1_ denoting the proportion of strains with different IgG antibody reactivity levels between the two groups. We set *μ*=12.5 for the remaining (100 − *s*) influenza strains that have the same IgG antibody reactivity levels between the two groups. For the first *s* influenza strains that have different IgG antibody reactivity levels between the first group and the second group, the mean differences Δ*μ* between the two groups were 1-2 with an equal increment amount of 1/*s*. For example, the increment amount was 1/25=0.04 when the first 25% influenza strains were different between groups (100 × 0.25=25).

The diagonal variables of the variance-covariance matrix Σ were all equal to 1 (denotes measurement errors from either continuous assays or titer-based assays), and off diagonal values were all 0.4 (denotes moderate correlations between influenza HA variants within the same influenza strain group). We assumed that the two groups have equal sample sizes of *n*, with *n*=5,10,20, and 30 over a range covering small, medium, and large sample size situations.

For simulations, we generated simulated data where HAI_*ijk*_ and MN_*ijk*_ titer values were derived from simulated mPLEX_*ijk*_ (*y*_*ijk*_) continuous values. We first generated the continuous data using one of the fitted linear regression models from the scatter plot between the logarithm (log_2_) of the titer data and the logarithm of the concentration data for the influenza A/Indonesia/5/05 strain determined by the mPLEX-Flu assay. We assumed *t*_*ijk*_ is the logarithm of the titer values of the IgG antibody reactivity levels from *i*th influenza virus strain, *j*th group, and *k*th sample (*i*=1,2,…, 100; *j*=1,2; *k*=1,2,…, *n*). First, we estimated the corresponding *t*_*ijk*_^*∗*^ value based on the relationship estimated from the regression model between the logarithm of the titer values and the logarithm of the concentration values, i.e.,

(3) $$\begin{array}{c} t_{ijk}^{*}=\beta_{0}+\beta_{1}\times y_{ijk}+\varepsilon_{ijk}, \end{array}$$

where *ε*_*ijk*_ ~ *N*(0,1). In our simulation studies, we set *β*_0_=−9.45 and *β*_1_=1.24 based on the fitted linear regression models between the logarithm of the titer data and the logarithm of the concentration data for the Hong Kong 97 influenza strain. After obtaining *t*_*ijk*_^*∗*^ values, we rounded down *t*_*ijk*_^*∗*^ values based on the measured values in the titer data to obtain the simulated titer data *t*_*ijk*_. The cutoff points we used in the simulation studies were (1.61, 1.96, 2.30, 2.65, 3.00, 3.34, 3.69, 4.04, 4.38, 4.73, 5.08, 5.42, 5.77, 6.11, 6.46, 6.69, 6.80, 7.15, 7.45, 7.80, 8.10, and 8.45). All *t*_*ijk*_^*∗*^ values within the intervals were rounded down to the lower bound of the interval, for example, *t*_*ijk*_^*∗*^ values in the interval [2.65, 3.00) are all equal to 2.65. For each influenza strain, the empirical Bayes method was used to test differences between groups through the *lmFit* and *eBayes* functions from the *limma* package in the statistical analysis software *R* [25, 26]. Each simulation study was repeated 1,000 times to obtain the estimated mean rejection, mean FDR, mean sensitivity, and mean specificity, using both the simulated concentration data and the titer data.

### 3.4. Simulation Results

**Figure 3:**
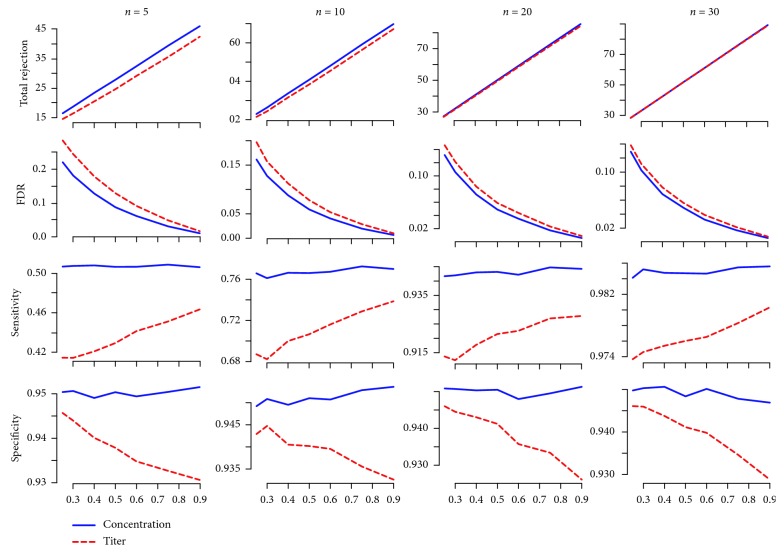
Simulation results comparing concentration data and titer data at sample sizes of 5, 10, 20, and 30 in each group at different *π*_1_ values.

Figure 3 shows the simulation results comparing the concentration data versus titer data for a sample size *n*=5 in each group. We found that more anti-HA IgG levels were identified as significantly different between vaccine groups when using the concentration data, as opposed to titer data. Consistent with these findings, FDRs using the concentration data were also relatively smaller than are those estimated using titer data. This was especially true when the proportion of strains that have different levels of IgG antibody binding between the two groups was small. Similarly, sensitivities calculated using the concentration data were much higher than the sensitivities estimated using the titer data (Table 2).

We also found that the sensitivities calculated using the concentration data were relatively more stable across different *π*_1_ values, whereas the sensitivities calculated from titer data increased with *π*_1_ (Figure 3). The specificities from analyses using the concentration data were also higher than the specificities derived from the titer data. Similarly, the specificities calculated using the concentration data were relatively more stable than the specificities calculated by using the titer data, which decreased with the increase of the proportion of strains that were different between groups. It is noticeable that the highest sensitivity was approximately 0.5 when the sample size was small (*n*=5 in each group), whereas the specificity was relatively high, with the lowest specificity being >0.93.

Similar trends were identified when we further increased the sample sizes in each group to *n*=10, 20, and 30 (Figure 3). Both sensitivities and specificities were higher when estimated using concentration data instead of titer data (Table 2). The total numbers of identified sera samples with significant differences between anti-HA IgG concentrations varied across strains. Significant differences were also present between the different groups. Both differences tended to converge as the sample size in each group increased from *n*=5 to *n*=30. However, the differences in estimated FDRs between those calculated from concentration versus titer data decreased as the sample sizes increased from 5 to 30 in each group.

## 4. Case Study

**Figure 4:**
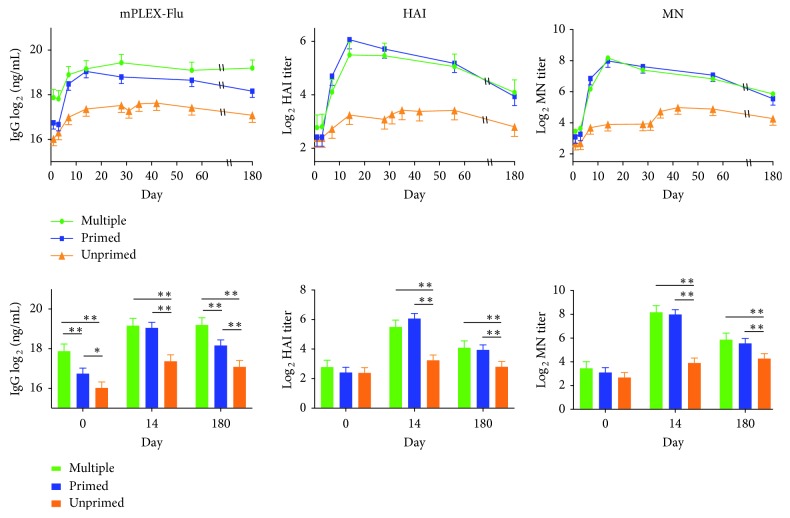
Longitudinal concentration or titer data of antibodies against influenza A/Indonesia/5/05 (vaccine strain for DMID 08-0059 study, a H5 vaccination clinical study). The linear mixed model tested for three vaccine groups. The approximate *t*-test in the linear mixed model is used for pairwise comparisons (^*∗∗*^ *p* ≤ 0.001; ^*∗*^ *p* ≤ 0.01).

We next compared the differences between the actual continuous antibody concentration data generated by the mPLEX-Flu assay with the titer data from the HAI and MN assays for all three vaccination strain-specific antibodies using the same serum samples from the DMID 08-0059 study [20]. The samples were collected longitudinally from three immunization groups: multiple primed, primed, and unprimed groups (see Table 1). There were 93 subjects: 16 (17.2%) in the multiple primed group, 46 (49.5%) in the primed group, and 31 (33.3%) in the unprimed group. The serum samples of both the multiple primed and primed groups were collected on day 0, day 7, day 14, day 28, day 56, and day 180/208. Samples from the unprimed group were collected on day 0, day 7, day 14, and day 28 (at which time the second booster vaccination was administered) and thereafter on day 31 (postboost day 3), day 35 (postboost day 7), day 42 (postboost day 14), day 56 (postboost day 28), and day 180/208. The results from the mPLEX-Flu and HAI/MN assays are shown in Figure 4.

We fit a linear mixed effects model on log-transformed concentration and HAI/MN data to examine the group differences at each time point:

(4) $$\begin{array}{c} Y=X\beta +Z\mu +\varepsilon , \end{array}$$

where *Y* denotes the vector of observation with *E*(*Y*)=*Xβ*; *β* denotes the unknown vector of fixed effects of group, time points, interaction between group and time points, and covariates such as age at enrollment, gender, ethnicity (White or non-White), dose (two dose levels: 15 and 90 *µ*g), and batch (five batches); *μ* is the unknown vector of random effects due to repeated measurements from the same subject, with mean *E*(*μ*)=0 and variance-covariance matrix var(*μ*)=*G*, where we assume *G* equals an autoregressive 1 variance-covariance matrix; and *ϵ* denotes random errors with *E*(*ε*)=0 and *var*(*ε*)=*R*, which is an identity matrix. The comparisons between groups at different time points were conducted using the linear contrast approach within the linear mixed models [27].

We applied the same linear mixed model to both continuous concentration data and titer data from the influenza vaccine strain A/Indonesia/5/05 and checked the distribution of residuals from the linear mixed models. The histogram and QQ-plot of the residuals showed approximate normal distribution of the log-transformed concentration data and the titer data (Figure 2). The results of the residuals from using the titer data were less normally distributed than the residuals using the concentration data, which is to be expected, as the titer data were more discrete than the concentration data. The overall estimated mean difference in vaccine antibody levels was significant between the multiple primed group and the primed group from the concentration data, whereas the differences were not significant from the titer data (Table 3). Meanwhile, significant differences were observed between the three groups at the baseline when we used concentration data from the mPLEX-Flu assay, whereas no significant differences were observed between the three groups when we used titer data from either the HAI or the MN assays (Figure 4). Further, the difference between the multiple primed group and the single-primed group at 180 days was significant when analyzing concentration data, but not significant using the titer data. The estimated differences at other time points were consistent between the concentration data and titer data.

## 5. Discussion

The HAI assay has been used for over 70 years as the gold standard assay to estimate the antibodies that specifically bind with the sialic acid binding site of HA on the surfaces of influenza viruses. Traditional titering-based assays like HAI do have some advantages, such as simplicity, cost, ease of use, and a straightforward statistical analysis method (geometric mean comparisons). For this reason, the HAI antibody titers of ferret antiserum from infected and vaccinated animals are still used to provide data for calculating the antigenic distances between current influenza virus strains and by the World Health Organization (WHO) to determine the strains for each year's influenza vaccine [9, 28, 29]. Recent studies have shown that influenza virus vaccine responses may critically depend on existing anti-HA immunity from prior influenza infection and/or vaccination [5, 6], which may be very hard to assess using any of the traditional single-dimensional assays, such as HAI, MN, and ELISA. Our studies [15–19] have previously demonstrated that the mPLEX-Flu assay allows for efficient assessment of antibody responses covering the HAs of over 50 previous and current circulating and vaccine strains. Thus, the assay provides a high-throughput and quantitative estimate of the imprinting pattern for each subject both before and after vaccination. In addition, we found that there were several significant differences between groups identified by using the mPLEX-Flu assay but could not be detected using the HAI and MN assay in this H5 vaccine clinical study.

In this study, we directly compared the statistical conclusions reached from analyzing influenza vaccine-specific antibody responses using semiquantitative (HAI and MN) vs. continuous assays (mPLEX-Flu). Our simulation studies showed the superiority of the continuous assays to the semiquantitative assays, as indicated by higher sensitivity, higher specificity, and lower FDR values in vaccine experimental group comparisons. This indicated that the continuous readout mPLEX-Flu assay enhanced statistical discernment when analyzing for differences between experimental groups in clinical vaccine studies. Compared to titering assays, the continuous readout (mPLEX-Flu) assay generates data that are more normally distributed after log transformation. Thus, continuous readout assays can provide more consistent results, with more depth than titering assays.

Furthermore, we also directly compared the antibody data from those different assays in an anti-H5 influenza vaccine study [20]. When using data from the continuous assay (mPLEX-Flu), we found several significant differences between vaccine experimental groups that had been deemed statistically insignificant when analyzed using data from semiquantitative assays (see Table 3). For example, analysis of the mPLEX-Flu data showed that the antibody levels of the H5 multiple primed group were statistically significantly higher than those of either the primed or unprimed groups. In addition, the anti-influenza antibody levels of the primed group were also higher than those of the unprimed group before vaccination (day 0). These results are also consistent with the increased antibody levels of the multiple primed group compared with the primed group and with the primed group compared with the unprimed group 180 days after vaccination, also different from the conclusions reached when analyzing HAI and MN data.

The above results are particularly important when clinical trials are conducted to compare vaccine efficacy considering either HA seniority or imprinting for different flu exposure histories. Type-II errors and elevated FDRs are more likely to happen when analyzing titering-based assay data, and they may result in the mistaken conclusion that there is no difference in vaccine efficacy between groups. In contrast, our work strongly suggests that continuous assays have fewer Type-II errors and are specifically useful when comparing antibody binding differences in clinical vaccine trials, especially when evaluating the persistence of vaccine-induced immunity after longer postvaccination intervals (i.e., 3 or ≥6 months). The rounding issues are especially important when comparing the efficacy of vaccine groups in clinical trials.

Some caveats apply to this analysis. Our simulation studies assumed that anti-HA IgG antibody levels followed a multivariate normal distribution with moderate correlations among multiple vaccine strains. This assumption was based on the distribution of the experimental data obtained from the continuous mPLEX-Flu assays. As other vaccines may target viral proteins that have more strain-to-strain heterogeneity, this assumption may not be necessary in such cases. Next, the titer data were generated based on the association between the logarithm of the titer data and the logarithm of the concentration data for one influenza strain. However, we expect similar simulation results across different influenza strains due to the semiquantitative characteristics of titer data and the continuous characteristics of concentration data. Finally, we did not test other continuous readout assays (e.g., ELISA); thus, specific assay characteristics might limit the generalizability of these findings.

In conclusion, this work suggests that the mPLEX-Flu continuous assay is superior to titering assays (e.g., HAI and MN) when comparing the effectiveness of treatment groups in influenza vaccine studies. This appears to be the case not only due to the multidimensional data generated by the mPLEX-Flu assay but also because statistical analysis using the continuous antibody concentration data results in improved precision and statistical discrimination between treatment groups. These findings will be critical for the design of future influenza vaccine trials and clinical studies. Finally, these results are likely generalizable to other fields that currently use titer-based assays for between-group statistical comparisons where continuous readout assays are available.

## Figures and Tables

**Table 1:** Vaccination strategy of the DMID 08-0059 study.

		Days after vaccination with inactivated A/Indonesia/5/05 (A/Ind05)							
Group	*n*	0	7	14	28	35	42	56	180/208
Primed	46	✕	✕	✕	✕			✕	✕
Multiple boost	16	✕	✕	✕	✕			✕	✕
Unprimed	31	✕	✕	✕	✕	✕	✕	✕	✕

Note: “✕” denotes that blood samples were collected before vaccination (day 0) and on days 7, 14, 28, 56, and 180 after vaccination. For the unprimed group, blood samples were collected before vaccination (day 0), on days 7, 14, and 28 before boosting, on day 28, and then on days 7 (day 35), 14 (day 42), 28 (day 56), and 180 (day 208) after boosting. Both the primed and the multiple boost groups had received the inactivated subvirion influenza A/Vietnam/1203/04 (A/Vie04) vaccine in 2005-2006. In addition, the multiple primed group also had received the baculovirus expressed recombinant influenza A/Hong Kong/156/97 vaccine (A/HK97) in 1997-1998. The unprimed group received only the A/Ind05 vaccine and a second booster vaccination at 28 days.

**Table 2:** Sensitivity and specificity using the concentration data (Con.) vs. titer data (Tit.).

Sample size	*π*_1_	Con.	Tit.	Con.	Tit.
		Sensitivity		Specificity	
*n*=5	0.25	0.5067	0.4151	0.9503	0.9456
	0.30	0.5074	0.4150	0.9505	0.9439
	0.40	0.5079	0.4216	0.9490	0.9400
	0.50	0.5065	0.4299	0.9503	0.9378
	0.60	0.5065	0.4420	0.9493	0.9348
	0.75	0.5087	0.4517	0.9503	0.9326
	0.90	0.5061	0.4639	0.9514	0.9306

*n*=10	0.25	0.7658	0.6871	0.9492	0.9429
	0.30	0.7611	0.6823	0.9508	0.9447
	0.40	0.7663	0.6999	0.9495	0.9405
	0.50	0.7660	0.7065	0.9510	0.9402
	0.60	0.7673	0.7160	0.9507	0.9396
	0.75	0.7725	0.7288	0.9528	0.9356
	0.90	0.7700	0.7386	0.9536	0.9326

*n*=20	0.25	0.9417	0.9137	0.9509	0.9460
	0.30	0.9420	0.9124	0.9507	0.9445
	0.40	0.9430	0.9177	0.9503	0.9430
	0.50	0.9432	0.9215	0.9505	0.9412
	0.60	0.9422	0.9226	0.9480	0.9358
	0.75	0.9447	0.9269	0.9495	0.9334
	0.90	0.9442	0.9278	0.9513	0.9261

*n*=30	0.25	0.9841	0.9737	0.9498	0.9461
	0.30	0.9852	0.9746	0.9503	0.9460
	0.40	0.9848	0.9754	0.9506	0.9438
	0.50	0.9847	0.9760	0.9484	0.9411
	0.60	0.9847	0.9765	0.9501	0.9398
	0.75	0.9855	0.9783	0.9478	0.9346
	0.90	0.9856	0.9803	0.9469	0.9288

**Table 3:** Clinical influenza vaccine study group comparisons using data from mPLEX-Flu, HAI, and MN assays.

Day	Group comparison	mPLEX-Flu		HAI		MN	
		Δ (SE)	*p* value	Δ (SE)	*p* value	Δ (SE)	*p* value
0	Multiple vs. primed	0.78 (0.21)	0.0002^*∗∗*^	0.25 (0.29)	0.4003	0.25 (0.34)	0.4658
	Multiple vs. unprimed	1.28 (0.23)	<0.0001^*∗∗*^	0.27 (0.31)	0.3912	0.54 (0.36)	0.1362
	Primed vs. unprimed	0.50 (0.19)	0.0109	0.02 (0.23)	0.9298	0.29 (0.26)	0.2698

7	Multiple vs. primed	0.29 (0.21)	0.1831	−0.41 (0.30)	0.1648	−0.47 (0.34)	0.1736
	Multiple vs. unprimed	1.32 (0.25)	<0.0001^*∗∗*^	0.96 (0.31)	0.0024^*∗*^	1.72 (0.36)	<0.0001^*∗∗*^
	Primed vs. unprimed	1.04 (0.21)	<0.0001^*∗∗*^	1.37 (0.23)	<0.0001^*∗∗*^	2.19 (0.26)	<0.0001^*∗∗*^

14	Multiple vs. primed	0.08 (0.21)	0.7010	−0.40 (0.29)	0.1807	0.13 (0.34)	0.7005
	Multiple vs. unprimed	1.25 (0.25)	<0.0001^*∗∗*^	1.56 (0.31)	<0.0001^*∗∗*^	2.97 (0.36)	<0.0001^*∗∗*^
	Primed vs. unprimed	1.17 (0.21)	<0.0001^*∗∗*^	1.95 (0.23)	<0.0001^*∗∗*^	2.84 (0.26)	<0.0001^*∗∗*^

28	Multiple vs. primed	0.44 (0.22)	0.0483	−0.17 (0.29)	0.5571	−0.15 (0.34)	0.6559
	Multiple vs. unprimed	1.32 (0.25)	<0.0001^*∗∗*^	1.65 (0.31)	<0.0001^*∗∗*^	2.41 (0.36)	<0.0001^*∗∗*^
	Primed vs. unprimed	0.88 (0.21)	<0.0001^*∗∗*^	1.83 (0.23)	<0.0001^*∗∗*^	2.56 (0.27)	<0.0001^*∗∗*^

56	Multiple vs. primed	0.31 (0.21)	0.1388	−0.08 (0.29)	0.7819	−0.16 (0.34)	0.6306
	Multiple vs. unprimed	1.16 (0.24)	<0.0001^*∗∗*^	1.14 (0.31)	0.0003^*∗∗*^	1.35 (0.36)	0.0003^*∗∗*^
	Primed vs. unprimed	0.85 (0.21)	<0.0001^*∗∗*^	1.22 (0.23)	<0.0001^*∗∗*^	1.51 (0.27)	<0.0001^*∗∗*^

180	Multiple vs. primed	0.72 (0.20)	0.0006^*∗∗*^	0.10 (0.29)	0.7293	0.21 (0.34)	0.5351
	Multiple vs. unprimed	1.46 (0.24)	<0.0001^*∗∗*^	0.89 (0.32)	0.0050^*∗*^	1.10 (0.36)	0.0027^*∗*^
	Primed vs. unprimed	0.75 (0.21)	0.0003^*∗∗*^	0.79 (0.23)	0.0008^*∗∗*^	0.89 (0.27)	0.0010^*∗∗*^

Overall mean	Multiple vs. primed	0.56 (0.18)	0.0021^*∗*^	0.04 (0.23)	0.8501	0.13 (0.28)	0.6547
	Multiple vs. unprimed	1.31 (0.20)	<0.0001^*∗∗*^	0.88 (0.24)	0.0003^*∗∗*^	1.30 (0.29)	<0.0001^*∗∗*^
	Primed vs. unprimed	0.75 (0.17)	<0.0001^*∗∗*^	0.84 (0.17)	<0.0001^*∗∗*^	1.17 (0.20)	<0.0001^*∗∗*^

*Note.* Linear mixed models were used to fit the data from the mPLEX-Flu, HAI, and MN assays with adjustment for dose, age at enrollment, gender, ethnicity, and batches. Pairwise comparisons were used to compare overall group differences and group differences at each day for the three vaccine groups (Δ means estimated differences between groups, SE means standard errors associated with Δ, ^*∗∗*^ *p* ≤ 0.001, and ^*∗*^ *p* ≤ 0.01).

## Data Availability

The HAI, MN, and mPLEX-Flu experimental data used to support the findings of this study are included within the supplementary information files.

## Supplementary Materials

Supplementary 1 S1 data: R code used for simulation and generating the simulated data for continuous and titering assays.

Supplementary 2 S2 data: actual HAI, MN, and mPLEX-Flu experimental data.
