# Supplementary material for: Continuous Readout versus Titer-Based Assays of Influenza Vaccine Trials: Sensitivity, Specificity, and False Discovery Rates
Source: Comput Math Methods Med. 2019 May 8;2019:9287120. doi: 10.1155/2019/9287120 (PMC6530215; doi:10.1155/2019/9287120)
Supplement: Supplementary 1 — S1 data: R code used for simulation and generating the simulated data for continuous and titering assays. [file 9287120.f1.pdf]

```
## Generate simulation data following lognormal disribution

num <- 1
total <- 1000
diffpro <- c(0.25, 0.30, 0.40, 0.50, 0.60, 0.75, 0.90)
nmeasure <- 100
ndiff <- nmeasure *diffpro
## For different sample size the samplesize values has been changed to
20,  40, and 60.
samplesize <- 10

## For different sample size the samplesize values has been changed to
10,  20, and 30.
trtsize <- 5
r <- 0.4
a <- sqrt((1-r)/(1+r))
library(limma)

set.seed(1234)

alpha  <- 0.05

ttestp_con <- ttestp_tit <- rep(NA, nmeasure)

totRejection_con <- FDR_con <- Power_con <- sensitivity_con <-
specificity_con <- matrix(NA, nrow = length(diffpro), ncol = total)

totRejection_tit <- FDR_tit <- Power_tit <- sensitivity_tit <-
specificity_tit <- matrix(NA, nrow = length(diffpro), ncol = total)



for (j in 1:length(diffpro))
{
 num <- 1
 diffmu <- seq(from = 1, to = 2, length.out = ndiff[j])
 while (num <= total)
 {
   group1 <- matrix(rnorm(nmeasure*(samplesize/2), 12.5, 1) +
a*rnorm(nmeasure*(samplesize/2), 0, 1), nmeasure, (samplesize/2))
   group2 <- matrix(rnorm(nmeasure*(samplesize/2), 12.5, 1) -
a*rnorm(nmeasure*(samplesize/2), 0, 1), nmeasure, (samplesize/2))
   y <- cbind(group1, group2)
   rownames(y) <- paste("Subject", 1:nmeasure)
   y[1:ndiff[j], (trtsize+1):samplesize] <- y[1:ndiff[j],
(trtsize+1):samplesize] + diffmu
   titer <- -9.45 + 1.24*y + rnorm(1)

   titer[titer < 1.61] <- 1.00
   titer[titer >= 1.61 & titer < 1.96] <- 1.61
```

```
   titer[titer >= 1.96 & titer < 2.30] <- 1.96
   titer[titer >= 2.30 & titer < 2.65] <- 2.30
   #titer[titer >= 2.48 & titer < 2.65] <- 2.48
   titer[titer >= 2.65 & titer < 3.00] <- 2.65
   titer[titer >= 3.00 & titer < 3.34] <- 3.00
   #titer[titer >= 3.23 & titer < 3.34] <- 3.23
   titer[titer >= 3.34 & titer < 3.69] <- 3.34
   titer[titer >= 3.69 & titer < 4.04] <- 3.69
   #titer[titer >= 3.86 & titer < 4.04] <- 3.86
   titer[titer >= 4.04 & titer < 4.38] <- 4.04
   #titer[titer >= 4.15 & titer < 4.38] <- 4.15
   titer[titer >= 4.38 & titer < 4.73] <- 4.38
   #titer[titer >= 4.56 & titer < 4.73] <- 4.56
   titer[titer >= 4.73 & titer < 5.08] <- 4.73
   titer[titer >= 5.08 & titer < 5.42] <- 5.08
   titer[titer >= 5.42 & titer < 5.77] <- 5.42
   titer[titer >= 5.77 & titer < 6.11] <- 5.77
   titer[titer >= 6.11 & titer < 6.46] <- 6.11
   titer[titer >= 6.46 & titer < 6.69] <- 6.46
   titer[titer >= 6.69 & titer < 6.80] <- 6.69
   titer[titer >= 6.80 & titer < 7.15] <- 6.80
   titer[titer >= 7.15 & titer < 7.45] <- 7.15
   titer[titer >= 7.45 & titer < 7.80] <- 7.45
   titer[titer >= 7.80 & titer < 8.10] <- 7.80
   titer[titer >= 8.10 & titer < 8.45] <- 8.10
   titer[titer >= 8.45] <- 8.45

   label <- c(rep(0, samplesize - trtsize), rep(1, trtsize))
   design <- model.matrix(~1+label)

   ## Use concentration data for the test
   fit <- lmFit(y, design, na.rm = TRUE)
   fit <- eBayes(fit)
   eBpval <- fit$p.value[, 2]
   eBadjp <- p.adjust(eBpval, method = "BH")
   eBadjp <- eBpval

   #for (i in 1:nmeasure)
   #{
   #  ttest_con <- t.test(y[i, label == 0], y[i, label == 1], na.rm =
TRUE)
   #  ttestp_con[i] <- ttest_con$p.value
   #}

   #eBadjp <- ttestp_con

   totRejection_con[j, num] <- sum(eBadjp <= alpha, na.rm = TRUE)
   FDR_con[j, num] <- sum(eBadjp[(ndiff[j]+1): nmeasure] <= alpha,
na.rm = TRUE)/totRejection_con[j, num]
   Power_con[j, num] <- sum(eBadjp[1:ndiff[j]] <= alpha, na.rm =
```

```
TRUE)/ndiff[j]
   sensitivity_con[j, num] <- sum(eBadjp[1:ndiff[j]] <= alpha, na.rm =
TRUE)/ndiff[j]
   specificity_con[j, num] <- sum(eBadjp[(ndiff[j]+1): nmeasure] >
alpha, na.rm = TRUE)/(nmeasure - ndiff[j])

   ## Use titer data for the test
   fit_tit <- lmFit(titer, design, na.rm = TRUE)
   fit_tit <- eBayes(fit_tit)
   eBpval_tit <- fit_tit$p.value[, 2]
   eBadjp_tit <- p.adjust(eBpval_tit, method = "BH")
   eBadjp_tit <- eBpval_tit

   #for (i in 1:nmeasure)
   #{
    # ttest_tit <- t.test(titer[i, label == 0], titer[i, label == 1],
na.rm = TRUE)
    # ttestp_tit[i] <- ttest_tit$p.value
   #}

   #eBadjp_tit <- ttestp_tit

   totRejection_tit[j, num] <- sum(eBadjp_tit <= alpha, na.rm = TRUE)
   FDR_tit[j, num] <- sum(eBadjp_tit[(ndiff[j]+1): nmeasure] <= alpha,
na.rm = TRUE)/totRejection_tit[j, num]
   Power_tit[j, num] <- sum(eBadjp_tit[1:ndiff[j]] <= alpha, na.rm =
TRUE)/ndiff[j]
   sensitivity_tit[j, num] <- sum(eBadjp_tit[1:ndiff[j]] <= alpha,
na.rm = TRUE)/ndiff[j]
   specificity_tit[j, num] <- sum(eBadjp_tit[(ndiff[j]+1): nmeasure] >
alpha, na.rm = TRUE)/(nmeasure - ndiff[j])

   num <- num + 1

   }
}

meanRej_con <- meanFDR_con <- meanPower_con <- meanSensitivity_con <-
meanSpecificity_con <- rep(NA, length(diffpro))

meanRej_tit <- meanFDR_tit <- meanPower_tit <- meanSensitivity_tit <-
meanSpecificity_tit <- rep(NA, length(diffpro))

for (i in 1:length(diffpro))
{
  meanRej_con[i] <- mean(totRejection_con[i, ], na.rm = TRUE)
  meanFDR_con[i] <- mean(FDR_con[i, ], na.rm = TRUE)
  meanPower_con[i] <- mean(Power_con[i, ], na.rm = TRUE)
  meanSensitivity_con[i] <- mean(sensitivity_con[i, ], na.rm = TRUE)
  meanSpecificity_con[i] <- mean(specificity_con[i, ], na.rm = TRUE)
```

```
  meanRej_tit[i] <- mean(totRejection_tit[i, ], na.rm = TRUE)
  meanFDR_tit[i] <- mean(FDR_tit[i, ], na.rm = TRUE)
  meanPower_tit[i] <- mean(Power_tit[i, ], na.rm = TRUE)
  meanSensitivity_tit[i] <- mean(sensitivity_tit[i, ], na.rm = TRUE)
  meanSpecificity_tit[i] <- mean(specificity_tit[i, ], na.rm = TRUE)
}

print(cbind(meanRej_con, meanRej_tit))
print(cbind(meanFDR_con, meanFDR_tit))
print(cbind(meanPower_con, meanPower_tit))
print(cbind(meanSensitivity_con, meanSensitivity_tit))
print(cbind(meanSpecificity_con, meanSpecificity_tit))

simulation_results <- cbind(meanRej_con, meanRej_tit, meanFDR_con,
meanFDR_tit, meanPower_con,
meanPower_tit, meanSensitivity_con, meanSensitivity_tit,
meanSpecificity_con, meanSpecificity_tit)

write.csv(simulation_results, file = "/Users/dli3/Documents/
Influ_sim_results_n_10.csv")


#pdf(file="06072015n_30_cor_4_lnorm.pdf", width=4, height=4)
dev.off()
par(mfrow=c(2,2), cex=0.7, mar=c(4.5, 4.5, 1.5, 0.7))
g_range<-range(meanRej_con, meanRej_tit, na.rm = TRUE)
plot(diffpro, meanRej_con, type="l", lwd=2, col="blue", ylim=g_range,
ann=FALSE)
box()
lines(diffpro, meanRej_tit, type="l", lty=2, lwd=2, col="red")
title(xlab=expression(pi[1]))
title(ylab="Total Rejection")
legend(0.3, g_range[2], c("Concentration","Titer"), cex=0.8,
       col=c("blue","red"), lwd=2, lty=1:3, bty="n")
#text(1, g_range[2], "A")

g_range<-range(meanFDR_con, meanFDR_tit, na.rm = TRUE)
plot(diffpro, meanFDR_con, type="l", lwd=2, col="blue", ylim=g_range,
ann=FALSE)
box()
lines(diffpro, meanFDR_tit, type="l", lty=2, lwd=2, col="red")
title(xlab=expression(pi[1]))
title(ylab="FDR")
legend(0.6, g_range[2], c("Concentration","Titer"), cex=0.8,
       col=c("blue","red"), lwd=2, lty=1:3, bty="n")
#text(1, g_range[2], "B")

g_range<-range(meanSensitivity_con, meanSensitivity_tit, na.rm = TRUE)
plot(diffpro, meanSensitivity_con, type="l", lwd=2, col="blue",
```

```
ylim=g_range, ann=FALSE)
box()
lines(diffpro, meanSensitivity_tit, type="l", lty=2, lwd=2, col="red")
title(xlab=expression(pi[1]))
title(ylab="Sensitivity")
legend(0.3, 0.50, c("Concentration","Titer"), cex=0.8,
       col=c("blue","red"), lwd=2, lty=1:3, bty="n")
#text(1, g_range[2], "C")

g_range<-range(meanSpecificity_con, meanSpecificity_tit, na.rm = TRUE)
plot(diffpro, meanSpecificity_con, type="l", lwd=2, col="blue",
ylim=g_range, ann=FALSE)
box()
lines(diffpro, meanSpecificity_tit, type="l", lty=2, lwd=2, col="red")
title(xlab=expression(pi[1]))
title(ylab="Specificity")
legend(0.6, 0.948, c("Concentration","Titer"), cex=0.8,
       col=c("blue","red"), lwd=2, lty=1:3, bty="n")
#text(1, g_range[2], "D")

dev.off()

#box_data <- simulation_results
#colnames(box_data) <- c(rep(c("Concentration", "Titer"), 5))

#par(mfrow=c(2,2), cex=0.7, mar=c(4.5, 5, 1.5, 1.5))
#boxplot(box_data[, 1:2], col=c("blue", "red"), boxwex=0.5, xlab=NULL,
ylab="Total Rejection")
#boxplot(box_data[, 3:4], col=c("blue", "red"), boxwex=0.5, xlab=NULL,
ylab="FDR")
#boxplot(box_data[, 7:8], col=c("blue", "red"), boxwex=0.5, xlab=NULL,
ylab="Sensitivity")
#boxplot(box_data[, 9:10], col=c("blue", "red"), boxwex=0.5,
xlab=NULL, ylab="Specificity")

#par(mfrow=c(1,1), cex=0.7, mar=c(4.5, 5, 1.5, 1.5))
#plot(titer, y, xlab = "Titer", ylab = "Concentration")
```
